# Joint association of physical activity and the geriatric nutritional risk index with survival outcomes among cancer survivors in the United States: a population-based cohort study

**DOI:** 10.3389/fnut.2024.1483507

**Published:** 2024-12-30

**Authors:** Jing Wei, Qingyue Zeng, Ming Liu

**Affiliations:** ^1^Department of Medical Oncology, Gastric Cancer Center, West China Hospital, Sichuan University, Chengdu, China; ^2^General Practice Ward, International Medical Center Ward, General Practice Medical Center, National Clinical Research Center for Geriatrics, West China Hospital, Sichuan University, Chengdu, China

**Keywords:** cancer survivors, physical activity, nutritional status, geriatric nutrition risk index, mortality, cohort study

## Abstract

**Introduction:**

The relationship between physical activity (PA) and nutritional status on the prognosis of cancer survivors remains underexplored. We aimed to investigate the combined effects of PA and Geriatric Nutritional Risk Index (GNRI) on prognostic assessment of survival outcomes in US cancer survivors.

**Methods:**

2,619 subjects were screened from the National Health and Nutrition Examination Survey (NHANES) database from 1999 to 2018. The self-reported Global Physical Activity Questionnaire (GPAQ) for PA assessment, and the GNRI for nutritional status assessment. Kaplan–Meier (K-M) curves and Cox proportional risk models were used to evaluate the effect of PA combined with GNRI on the prognostic outcomes of death in cancer survivors.

**Results:**

The sufficient PA (≥600 MET min/week) combined with High-GNRI (>98) subgroups significantly reduced the risk of all-cause mortality (HR: 0.56; 95% CI, 0.35–0.90) and cancer-related mortality (HR: 0.24; 95% CI, 0.12–0.50) compared to other subgroups. Subgroup analyses indicated that the combination of sufficient PA and High-GNRI was associated with a significantly reduced all-cause and cancer-related mortality among specific groups-including individuals of female, patients with non-obesity-related cancers, and those with higher educational attainment. After excluding participants who died within the first 12 months of follow-up, sensitivity analyses confirmed the robustness of the association between PA and GNRI in predicting prognostic outcomes among cancer survivors.

**Conclusion:**

Our study shows that among U.S. cancer survivors, sufficient PA combined with High-GNRI is linked to reduced mortality. These findings emphasize the benefits of PA and nutritional status in improving prognosis and support the need for further studies to develop targeted interventions.

## 1 Introduction

Cancer remains a major global health challenge, and the growing number of cancer survivors underscores the increasing need for comprehensive survivorship care (1). Older cancer survivors (≥65 years) are particularly vulnerable due to reduced physical activity (PA), functional decline, and nutritional deterioration. These factors contribute to obesity and metabolic disorders, which are strongly associated with poorer survival outcomes (2–4). Recent researches highlight the pivotal role of PA and nutrition in survivorship care. These modifiable factors can mitigate chronic inflammation, metabolic dysfunction, and immune suppression-critical mechanisms driving adverse outcomes in cancer survivors (5–7). Understanding the interplay between PA and nutrition is crucial for developing targeted interventions to improve long-term survival and quality of life.

Physical activity plays a vital role in enhancing survival outcomes by mitigating obesity and metabolic disorders (3). It improves muscle mass, cardiorespiratory fitness, and metabolic regulation, while simultaneously reducing systemic inflammation and bolstering immune function. Collectively, these mechanisms alleviate risks associated with comorbidities and treatment-related complications (8–12). Despite its well-established benefits, PA levels in cancer survivors are often markedly lower than those of the general population, largely due to fatigue, comorbidities, and treatment-related side effects (13, 14). Regular PA has the potential to restore metabolic efficiency, enhance treatment tolerance, and improve overall health. Evidence indicates that engaging in moderate-intensity PA (≥600 MET-minutes/week) is associated with significant health benefits for survivors (15, 16). However, further research is needed to elucidate the impact of PA across diverse cancer populations.

Nutritional status is another critical determinant of survival outcomes in cancer survivors, particularly in older adults who are at heightened risk of malnutrition (17, 18). The Geriatric Nutritional Risk Index (GNRI) is a validated tool that combines serum albumin levels and body weight to assess nutritional risk (19–21). GNRI has demonstrated strong reliability in predicting survival outcomes, particularly in hospitalized populations, as well as in oncology patients (19, 22–25). For instance, GNRI predicts surgical outcomes and overall survival in individuals diagnosed with lung cancer. Low GNRI scores are associated with higher recurrence rates and poorer survival in gastrointestinal cancers. Compared to other nutritional assessment tools such as the Prognostic Nutritional Index (PNI) or the Subjective Global Assessment (SGA), GNRI offers significant advantages (26, 27). While PNI incorporates both serum albumin and total lymphocyte count, GNRI emphasizes physical parameters like weight, which may be more directly related to nutritional risk, particularly in the elderly population (28). The relationship between PA and nutritional status on the prognosis of cancer survivors remains underexplored. GNRI relies on objective parameters, such as serum albumin and weight, making it more practical for outpatient settings. Additionally, it provides a comprehensive measure of both nutritional and inflammatory status, which is especially relevant for cancer survivorship care.

Physical activity and GNRI may have complementary effects on survival outcomes in cancer survivors. PA can improve GNRI scores by stabilizing body weight, enhancing muscle mass, and increasing serum albumin levels, while also reducing systemic inflammation (29–35). This synergistic relationship underscores the importance of integrating PA and GNRI into survivorship care strategies. However, the combined prognostic value of these factors remains underexplored. Future studies should aim to quantify their joint effects and identify subgroups of survivors who may derive the greatest benefit from tailored interventions.

This study investigates the associations of PA and GNRI with all-cause, cancer-related, and non-cancer-related mortality in a nationally representative cohort of cancer survivors. By examining the combined effects of PA and GNRI, the findings aim to provide evidence-based recommendations for incorporating these modifiable factors into survivorship care guidelines.

## 2 Methods

### 2.1 Data sources and study population

The National Health and Nutrition Examination Survey (NHANES), conducted by the National Center for Health Statistics (NCHS), is a cross-sectional survey designed to assess the health and nutritional status of the U.S. population. NHANES employs a complex, stratified, multistage probability sampling design to ensure nationally representative samples. Detailed descriptions of its methodology and protocols are available in the literature (36, 37). Data collection includes demographic, dietary, and health-related interviews, as well as physical examinations and laboratory tests performed in mobile examination centers. All NHANES protocols were approved by the NCHS Research Ethics Review Board, with written informed consent obtained from all participants.

Although NHANES is a cross-sectional survey, it enables longitudinal analysis through linkage with the National Death Index (NDI), allowing researchers to examine long-term survival outcomes. This study utilized NHANES data from 1999 to 2018, linked to NDI mortality data. Participants aged 40 years or older were included, and sociodemographic characteristics, health status, lifestyle factors, and daily physical activity levels were analyzed to evaluate the associations between PA, nutritional status, and survival outcomes among cancer survivors.

### 2.2 PA assessment and GNRI assessment

PA was assessed using the Global Physical Activity Questionnaire (GPAQ), a validated tool developed by the World Health Organization (WHO) to measure PA across three domains-work, commuting, and leisure-and sedentary behavior (38). Weekly PA was converted into metabolic equivalent minutes (MET-min) following WHO guidelines, with sufficient PA defined as ≥600 MET-min/week (equivalent to 150 min of moderate-intensity or 75 min of vigorous-intensity activity per week) and insufficient PA as <600 MET-min/week.

Nutritional status was assessed using the GNRI, a validated tool initially developed to evaluate nutritional risk in older patients in hospital or clinical settings. The GNRI has been validated against clinical outcomes such as mortality, hospitalization rates, and complications in elderly populations. It is a tool designed to identify patients at risk of malnutrition-related complications by combining serum albumin levels and ideal body weight, which reflect nutritional status. However, its primary function is as a risk assessment tool, not a comprehensive nutritional assessment. It is specifically aimed at capturing the risk associated with malnutrition. The GNRI was calculated using the following formula: GNRI = (1.489 × serum albumin (g/L)) + (41.7 × actual weight (kg)/ideal weight (kg)). Actual weight refers to the current weight as measured (either self-reported, directly measured, or abstracted from medical records). Ideal weight was calculated based on the standard BMI of 22 kg/m^2^ as follows: Ideal weight (kg) = 22 × (height (m)^2^). In this study, GNRI scores were categorized as high risk (≤98) and low risk (>98), consistent with prior research and clinical guidelines (19). Originally developed for hospitalized older adults, GNRI has been validated in diverse populations, including community-dwelling individuals and epidemiological cohorts, supporting its use in assessing nutritional risk and survival outcomes in older cancer survivors (39, 40).

Based on PA and the GNRI (threshold: 98), participants were classified into four groups: (1) Insufficient PA and High-GNRI (IH): PA <600 MET-min/week, GNRI >98; (2) Insufficient PA and Low-GNRI (IL): PA <600 MET-min/week, GNRI ≤98; (3) Sufficient PA and High-GNRI (SH): PA ≥600 MET-min/week, GNRI >98; and (4) Sufficient PA and Low-GNRI (SL): PA ≥600 MET-min/week, GNRI ≤98. This classification enabled analysis of the combined effects of PA and nutritional status on health outcomes.

### 2.3 Covariate evaluation

Potential covariates were selected based on established associations between lifestyle factors and cancer survivor prognosis: (i) Sociodemographic characteristics, including age, sex (male or female), race/ethnicity (Mexican American, non-Hispanic white, non-Hispanic black, other Hispanic, and multiracial/other), education level (under high school, high school or equivalent, and above high school), household income-to-poverty ratio (low: ≤1.3, medium: >1.3 to ≤3.5, and high: >3.5), and marital status (married or other). (ii) Behavioral variables, such as alcohol consumption (former, heavy, mild, moderate, or never) and smoking status (former, never, or current smoker) (41). Body mass index (BMI: kg/m^2^) classified using standard guidelines: normal or underweight (≤24.9 kg/m^2^), overweight (25–29.9 kg/m^2^), and obese (≥30 kg/m^2^; WHO, 2000). (iii) Comorbidities, including hypertension, diabetes mellitus, hyperlipidemia, and cardiovascular disease, were identified through self-reports, standardized medical questionnaires, laboratory tests, and imaging findings.

### 2.4 Diagnosis of cancer

Cancer diagnosis data, including type and age at diagnosis, were collected through face-to-face interviews and extracted from the “Medical Conditions” section of the NHANES database. Cancer survivors were identified by the question: “*Have you ever been told by a doctor or other health professional that you have cancer or any other type of malignancy?*.” Those who responded “*yes*” were further asked: “*What kind of cancer have you been diagnosed with?*” and “*What year or age were you first diagnosed with cancer?*”

To further analyze the joint effects of PA and GNRI on survival, cancer types were divided into obesity-related and non-obesity-related groups based on existing literature. Obesity-related cancers included breast, hematological, colorectal, esophageal, brain, gallbladder, liver, kidney, pancreatic, stomach, ovarian, and uterine cancers; all others were classified as non-obesity-related.

### 2.5 Determination of mortality

Mortality data for this study were obtained from the NCHS mortality files, linked to the NDI, as of December 31, 2019. The underlying causes of death were classified using the International Statistical Classification of Diseases and Related Health Problems, Tenth Revision (*ICD-10*). The primary outcome of this study was all-cause mortality, while secondary outcomes included cancer-related mortality and non-cancer-related mortality. Cancer-related mortality was defined as deaths attributed to malignant neoplasm (*ICD-10 codes C00-C97*). Follow-up time was calculated in months, starting from the date of the participant’s first visit to the mHealth medical examination center until the date of death or the end of the follow-up period on December 31, 2019.

### 2.6 Statistical analysis

This study adhered to NHANES Analytical Guidelines, applying sample clustering, stratification, and weighting for national representativeness. Participants were categorized by PA and GNRI. Descriptive statistics included means ± standard deviations for normally distributed continuous variables (Student’s t-test), medians (interquartile ranges) for non-normally distributed variables (Kruskal-Wallis test), and weighted proportions (%) for categorical variables (design-adjusted chi-square tests). Kaplan–Meier analysis estimated survival probabilities across PA-GNRI subgroups, with differences assessed using the log-rank test. Cox proportional hazards models evaluated associations between PA, GNRI, and mortality outcomes (all-cause, cancer-related, and non-cancer-related), with four models: crude (unadjusted), Model 1 (adjusted for age and sex), Model 2 (additionally adjusted for ethnicity, education, income, and marital status), and Model 3, the fully adjusted model (further adjusted for BMI, smoking, alcohol use, hypertension, diabetes, hyperlipidemia, and cardiovascular disease). Schoenfeld residuals validated the proportional hazards assumption. Restricted cubic spline (RCS) models were applied to evaluate potential non-linear associations between physical activity (PA), GNRI, and mortality, visually representing log hazard ratios (log HR). The evaluation was conducted using 3 knots, placed at the 25th, 50th, and 75th percentiles of the data distribution, to allow for flexible modeling of the relationship. The choice of knots was based on standard recommendations for spline modeling, ensuring an appropriate fit for the data.

This study handled missing data effectively, with a maximum missing rate of 5.9% across all variables. Multiple imputation was performed using the “mice” package in R software, incorporating all covariates into the imputation model. The pooled results, calculated using Rubin’s Rules, were used for subsequent statistical analyses. Sensitivity analyses verified the robustness of findings by comparing results before and after imputation, as well as excluding participants who died within the first 12 months of follow-up. Stratified analyses explored subgroup consistency (e.g., age groups, cancer types). All analyses were conducted in R (version 4.3.1), with *p*-values <0.05 deemed significant.

## 3 Results

### 3.1 Baseline characteristics of the study population

**Figure 1:**
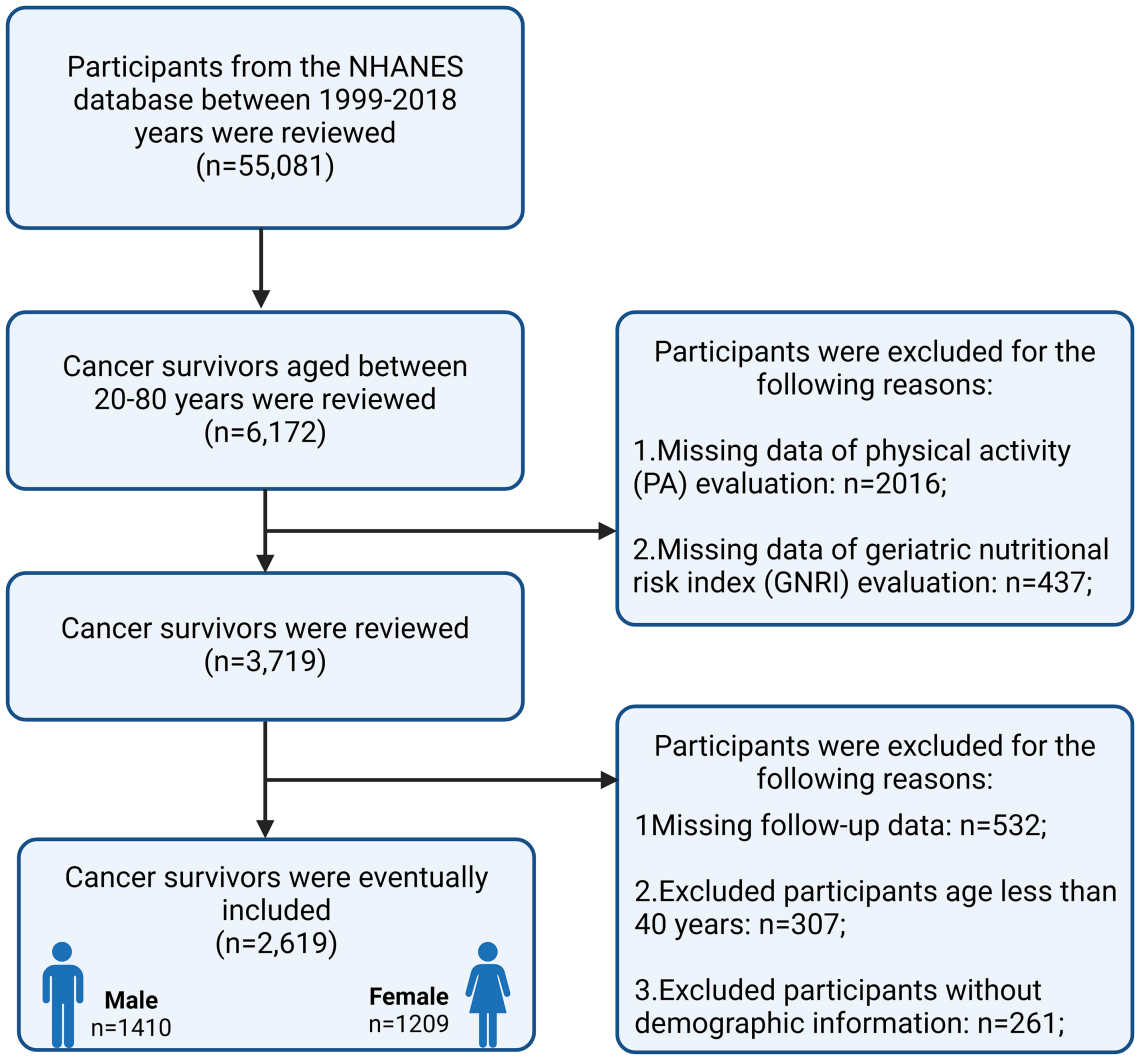
Flowchart of the selection strategy.

After screening the NHANES data for 10 cycles in this study, 2,619 individuals met the inclusion criteria and were finally included in the analysis. The specific screening process is detailed in Figure 1. Of all participants (weighted median age: 63.78 years, weighted percentage of females: 51.46%), 88.90% (*n* = 1914) were non-Hispanic Whites, 69.16% (*n* = 1,585) were college-educated, 69.54% (*n* = 1,684) were in a married status, 92.99% (*n* = 2,399) were in High-GNRI status, 66.83% (*n* = 1701) were in sufficiently active status, 34.08% (*n* = 892) had a BMI of > = 30 Kg/m^2^, 56.13% (*n* = 1,107) had families at high income levels. At the time of the study, more than the average patient was a moderate to heavy drinker (57.44%, *n* = 1,428), and 13.92% (*n* = 351) were still smoking. The prevalence of comorbidities was as follows: hypertension 58.04% (*n* = 1,675), diabetes mellitus (DM) 20.04% (*n* = 600), cardiovascular disease (CVD) 16.74% (*n* = 559) and hyperlipidaemia 80.61% (*n* = 2084). Overall, 64.94% (1701/2619) of cancer patients in the included population met PA recommendations (≥600 MET-min/week), and 91.60% (2,399/2619) were in High-GNRI status. Table 1 summarized the patient population details for the four different PA and GNRI level groups.

**Table 1:** Characteristics of the included cancer survivors from NHANES database.

Variable	Total	Insufficiently PA and High-GNRI group (IH) (*n* = 832)	Insufficiently PA and Low-GNRI group (IL) (*n* = 86)	sufficiently PA and High-GNRI group (SH) (*n* = 1,567)	sufficiently PA and Low-GNRI group (SL) (*n* = 134)	*P*-value
**Age, mean (SD)**	63.78 (0.31)	63.40 (0.54)	63.02 (1.71)	64.00 (0.36)	63.86 (1.48)	0.78
**Sex (%)**						0.21
Female	1,209 (51.46)	420 (54.12)	37 (40.97)	699 (50.86)	53 (48.31)	
Male	1,410 (48.54)	412 (45.88)	49 (59.03)	868 (49.14)	81 (51.69)	
**Ethnicity, *n* (%)**						0.001**
Mexican American	144 (1.52)	40 (1.16)	7 (2.11)	93 (1.64)	4 (2.04)	
Non-Hispanic Black	335 (4.53)	98 (4.43)	20 (9.76)	189 (4.06)	28 (8.72)	
Non-Hispanic White	1914 (88.90)	639 (90.46)	52 (82.75)	1,141 (88.95)	82 (80.85)	
Other Hispanic	120 (1.83)	23 (0.98)	5 (4.17)	82 (2.00)	10 (3.93)	
Other Race - including multi-racial	106 (3.22)	32 (2.97)	2 (1.20)	62 (3.35)	10 (4.45)	
**Educational level, *n* (%)**						0.76
Above high school	1,585 (69.16)	494 (67.89)	49 (62.49)	959 (70.20)	83 (67.40)	
High school or equivalent	596 (20.86)	197 (21.74)	19 (24.36)	354 (20.40)	26 (19.16)	
Under high school	438 (9.97)	141 (10.37)	18 (13.15)	254 (9.40)	25 (13.44)	
**Family_income, n (%)**						0.004**
High	1,107 (56.13)	335 (52.10)	32 (44.96)	697 (59.37)	43 (44.51)	
Low	492 (10.94)	175 (13.08)	17 (15.61)	265 (9.38)	35 (15.51)	
Medium	1,020 (32.93)	322 (34.82)	37 (39.43)	605 (31.25)	56 (39.97)	
**Marital status, *n* (%)**						0.12
Married	1,684 (69.54)	524 (68.46)	45 (55.11)	1,036 (70.53)	79 (72.37)	
Other	935 (30.46)	308 (31.54)	41 (44.89)	531 (29.47)	55 (27.63)	
**BMI, Kg/m** ^**2**^ **, mean (SD)**						< 0.001**
<24.9	775 (30.61)	229 (28.04)	31 (45.22)	451 (30.20)	64 (45.56)	
> = 30	892 (34.08)	305 (38.09)	21 (16.06)	531 (33.21)	35 (30.20)	
25 ~ 29.9	952 (35.31)	298 (33.87)	34 (38.72)	585 (36.59)	35 (24.24)	
**Alcohol.user, *n* (%)**						0.33
Former	590 (18.30)	207 (20.50)	25 (22.36)	321 (16.72)	37 (23.37)	
Heavy	208 (8.92)	66 (9.43)	5 (2.19)	126 (9.03)	11 (7.96)	
Mild	1,220 (48.52)	375 (45.39)	34 (43.87)	752 (50.11)	59 (50.65)	
Moderate	305 (15.47)	100 (16.38)	10 (16.23)	184 (15.40)	11 (9.48)	
Never	296 (8.79)	84 (8.30)	12 (15.35)	184 (8.75)	16 (8.55)	
**Smoke status, *n* (%)**						0.23
Former	1,130 (41.15)	378 (42.10)	34 (37.97)	657 (40.10)	61 (52.23)	
Never	1,136 (44.91)	344 (43.24)	34 (39.78)	705 (46.60)	53 (35.47)	
Now	351 (13.92)	109 (14.66)	18 (22.24)	204 (13.30)	20 (12.30)	
**Hypertension, *n* (%)**						0.37
No	944 (41.96)	269 (39.01)	29 (44.60)	597 (43.51)	49 (38.45)	
Yes	1,675 (58.04)	563 (60.99)	57 (55.40)	970 (56.49)	85 (61.55)	
**DM, *n* (%)**						0.3
No	2018 (79.94)	627 (78.76)	70 (85.95)	1,211 (79.92)	110 (85.23)	
Yes	600 (20.04)	205 (21.24)	16 (14.05)	356 (20.08)	23 (14.77)	
**Hyperlipidemia, *n* (%)**						< 0.0001***
No	535 (19.39)	151 (16.34)	47 (59.36)	267 (16.73)	70 (54.38)	
Yes	2084 (80.61)	681 (83.66)	39 (40.64)	1,300 (83.27)	64 (45.62)	
**CVD, *n* (%)**						0.45
No	2060 (83.26)	643 (81.83)	67 (78.82)	1,244 (83.99)	106 (85.74)	
Yes	559 (16.74)	189 (18.17)	19 (21.18)	323 (16.01)	28 (14.26)	
**PA_Group, *n* (%)**						< 0.0001***
Insufficiently active	918 (33.17)	832 (100.00)	86 (100.00)	0 (0.00)	0 (0.00)	
Sufficiently active	1701 (66.83)	0 (0.00)	0 (0.00)	1,567 (100.00)	134 (100.00)	
**GNRI_Group, *n* (%)**						< 0.0001***
Low-GNRI group	220 (7.01)	0 (0.00)	86 (100.00)	0 (0.00)	134 (100.00)	
High-GNRI group	2,399 (92.99)	832 (100.00)	0 (0.00)	1,567 (100.00)	0 (0.00)	

### 3.2 Correlation between PA, GNRI, and mortality

**Figure 2:**
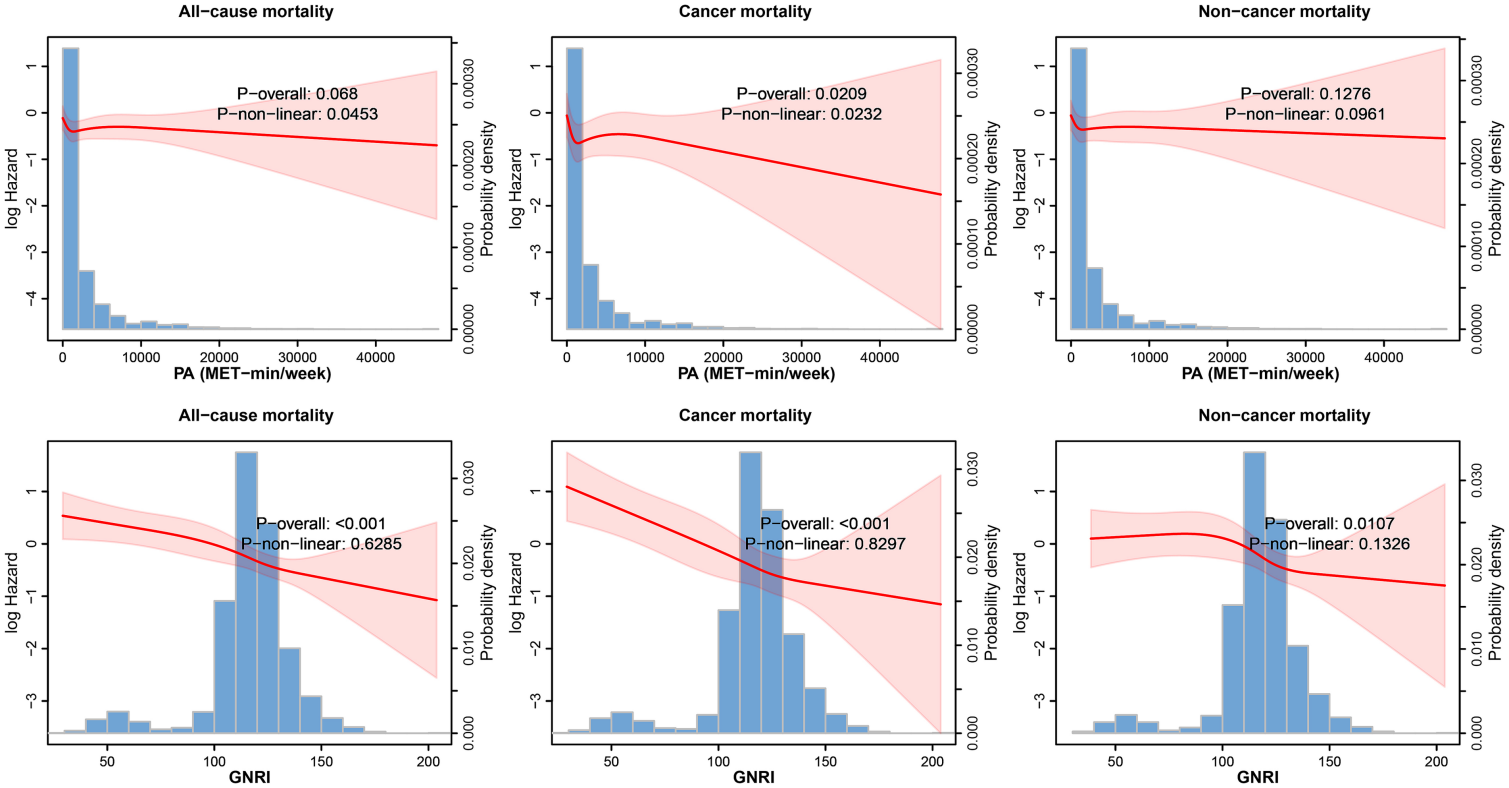
Restricted cubic spline analysis illustrating the non-linear associations between PA (MET-min/week), GNRI, and all-cause mortality, adjusted for key covariates. The shaded area represents the 95% confidence intervals.

During a median follow-up period of up to 7.83 year (interquartile interval: 4.25, 12.08), 846 deaths occurred, of which 279 were cancer-related and 567 were non-cancer-related. Figure 2 illustrates the dose–response relationships between PA, the GNRI, and mortality among cancer survivors. RCS analysis reveals non-linear associations: increasing PA reduces cancer-related mortality, while higher GNRI scores lower all-cause, cancer-related, and non-cancer-related mortality, particularly above the GNRI threshold of 98. These findings highlight the importance of sufficient PA and High-GNRI in improving survival outcomes for cancer survivors (Figure 2; Supplementary Figure S1). In crude models, cancer survivors with sufficient PA (≥600 MET-min/week) had an HR of 0.86 (95% CI: 0.72–1.03) for all-cause mortality, 0.72 (95% CI: 0.54–0.97) for cancer-related mortality, and 0.89 (95% CI: 0.72–1.09) for non-cancer-related mortality. Survivors with High-GNRI (>98) had an HR of 0.51 (95% CI: 0.37–0.71) for all-cause mortality, 0.32 (95% CI: 0.20–0.52) for cancer-related mortality, and 0.65 (95% CI: 0.45–0.96) for non-cancer-related mortality (Supplementary Table S1). In the fully adjusted model (Model 3), sufficient PA was associated with an HR of 0.82 (95% CI: 0.69–0.98) for all-cause mortality, 0.67 (95% CI: 0.50–0.91) for cancer-related mortality, and 0.82 (95% CI: 0.66–1.02) for non-cancer-related mortality. High-GNRI was associated with an HR of 0.61 (95% CI: 0.45–0.83) for all-cause mortality, 0.37 (95% CI: 0.22–0.63) for cancer-related mortality, and 0.79 (95% CI: 0.53–1.19) for non-cancer-related mortality (Supplementary Table S1).

### 3.3 Correlation between combined PA and GNRI and mortality

**Figure 3:**
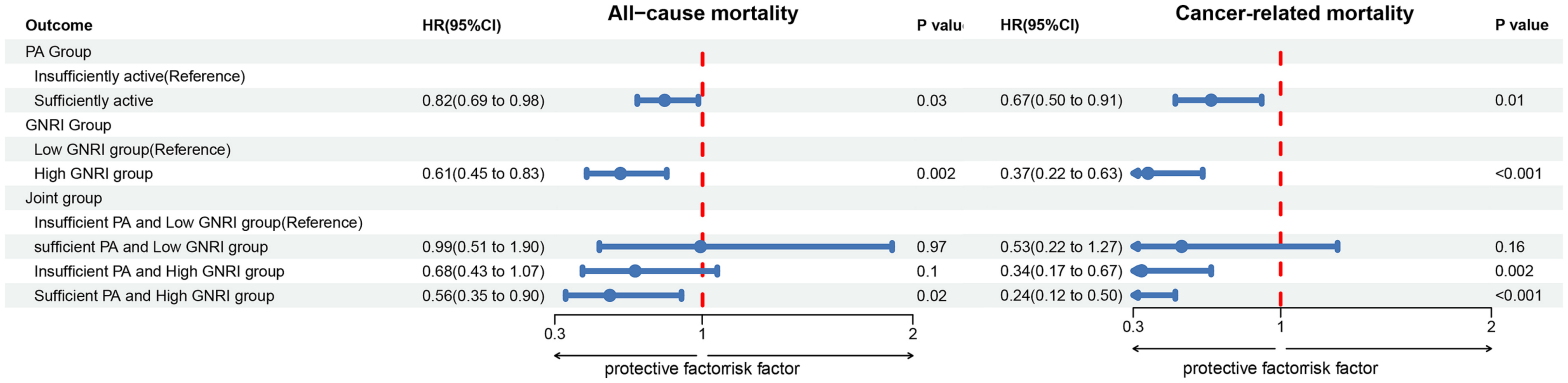
Forest plot illustrating the associations between PA, the GNRI, and their combination with all-cause and cancer-related mortality among 2,619 cancer survivors. The results are adjusted for potential confounders, including age, sex, race/ethnicity, marital status, education level, household income, BMI, smoking status, alcohol consumption, hypertension, diabetes, hyperlipidemia, and CVD. Insufficient PA: <600 MET-min/week; Sufficient PA: ≥600 MET-min/week; Low-GNRI: ≤98; High-GNRI: >98. *p < 0.05, **p < 0.01, ***p < 0.001 were considered statistically significant. PA, physical activity; GNRI, geriatric nutritional risk index; CVD, cardiovascular disease; HR, hazard ratio; CI, confidence interval.

**Figure 4:**
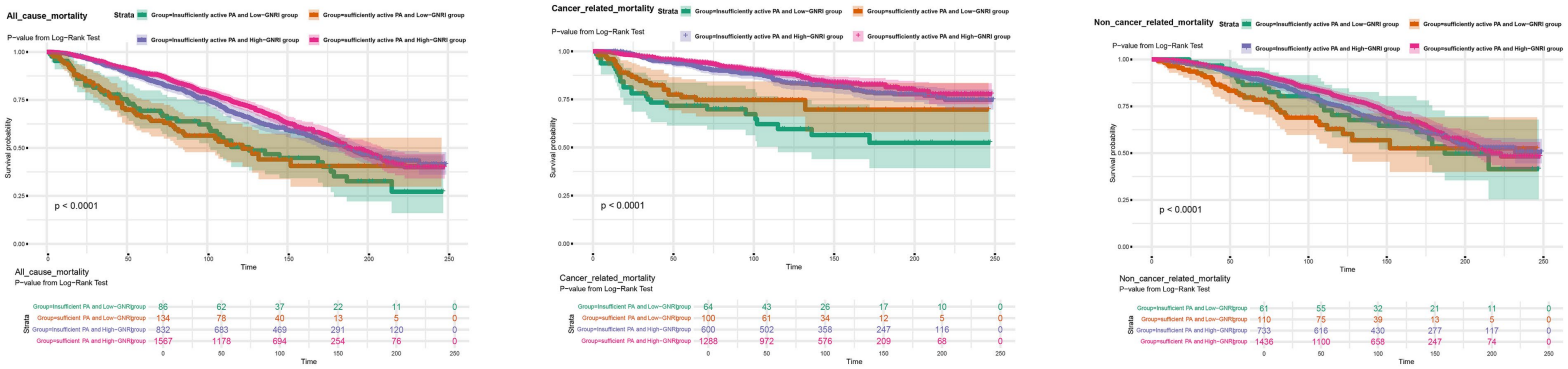
Kaplan–Meier survival curves illustrating overall survival across different PA and GNRI combination groups for all-cause mortality, cancer-related mortality, and non-cancer-related mortality. Statistical significance was denoted as follows: *p < 0.05; **p < 0.01; ***p < 0.001. PA, physical activity; GNRI, geriatric nutritional risk index.

In the joint analysis, the combination of sufficient PA (≥600 MET-min/week) and High-GNRI (>98) was associated with the lowest risk of all-cause, cancer-related, and non-cancer-related mortality. In the crude model, this group had an HR of 0.45 (95% CI: 0.27–0.73) for all-cause mortality, 0.21 (95% CI: 0.10–0.42) for cancer-related mortality, and 0.65 (95% CI: 0.35–1.18) for non-cancer-related mortality. After adjustment for age and sex in Model 1, the combination of sufficient PA and High-GNRI remained associated with the lowest mortality risk (Table 2). In the fully adjusted model (Model 3), the HR for all-cause mortality was 0.56 (95% CI: 0.35–0.90), for cancer-related mortality was 0.24 (95% CI: 0.12–0.50), and for non-cancer-related mortality was 0.86 (95% CI: 0.45–1.64) (Figure 3; Supplementary Figure S2). The p for trend values reflects the statistical significance of trends across PA and GNRI groups regarding mortality outcomes. Higher PA and GNRI levels show significant downward trends in all-cause and cancer-related mortality risks, while trends for non-cancer-related mortality are weaker, underscoring the need for further investigation to clarify these relationships (Table 2). Additionally, Kaplan–Meier survival curves demonstrated that the group with sufficient PA and High-GNRI had the highest overall survival compared to other groups (Figure 4).

**Table 2:** Association of PA combined with GNRI with all-cause mortality, cancer-related mortality, and non-cancer-related mortality among cancer survivors in the United States.

Mortality outcome	Crude model		Model 1		Model 2		Model 3	
	95%CI	*P*	95%CI	*P*	95%CI	*P*	95%CI	*P*
All-mortality								
PA + GNRI_Group								
Insufficient PA and Low-GNRI group	Ref		Ref		Ref		Ref	
sufficient PA and Low-GNRI group	0.85 (0.46,1.55)	0.59	0.90 (0.51,1.57)	0.70	1.02 (0.55,1.88)	0.96	0.99 (0.51,1.90)	0.97
Insufficient PA and High-GNRI group	0.51 (0.32,0.82)	0.01*	0.52 (0.34,0.79)	0.003**	0.60 (0.37,0.98)	0.04*	0.68 (0.43,1.07)	0.10
sufficient PA and High-GNRI group	0.45 (0.27,0.73)	0.001**	0.40 (0.26,0.62)	<0.0001***	0.48 (0.29,0.80)	0.004**	0.56 (0.35,0.90)	0.02*
p for trend (character 2 integer)		<0.001		<0.0001		<0.0001		<0.001
Cancer-mortality								
PA + GNRI_Group								
Insufficient PA and Low-GNRI group	Ref		Ref		Ref		Ref	
sufficient PA and Low-GNRI group	0.49 (0.22,1.09)	0.08	0.55 (0.24,1.25)	0.15	0.66 (0.29,1.49)	0.32	0.53 (0.22,1.27)	0.16
Insufficient PA and High-GNRI group	0.26 (0.13,0.51)	<0.0001***	0.28 (0.15,0.52)	<0.0001***	0.35 (0.18,0.69)	0.003**	0.34 (0.17,0.67)	0.002**
sufficient PA and High-GNRI group	0.21 (0.10,0.42)	<0.0001***	0.19 (0.10,0.36)	<0.0001***	0.25 (0.12,0.49)	<0.0001***	0.24 (0.12,0.50)	<0.001**
*p* for trend (character 2 integer)		<0.0001		<0.0001		<0.0001		<0.001
Non-cancer-mortality								
PA + GNRI_Group								
Insufficient PA and Low-GNRI group	Ref		Ref		Ref		Ref	
sufficient PA and Low-GNRI group	1.09 (0.55,2.19)	0.80	1.19 (0.61,2.30)	0.61	1.47 (0.68,3.18)	0.33	1.47 (0.63,3.42)	0.37
Insufficient PA and High-GNRI group	0.74 (0.41,1.32)	0.31	0.77 (0.45,1.32)	0.34	0.93 (0.49,1.76)	0.81	1.10 (0.57,2.11)	0.78
sufficient PA and High-GNRI group	0.65 (0.35,1.18)	0.15	0.57 (0.33,0.98)	0.04*	0.70 (0.37,1.33)	0.28	0.86 (0.45,1.64)	0.64
*p* for trend (character 2 integer)		0.03		<0.0001		0.005		0.05

The crude model represents unadjusted analyses.

Model 1: Adjusted for age and sex.

Model 2: Adjusted for age, sex, ethnicity, family income, education, and marital status.

Model 3: Adjusted for age, sex, ethnicity, marital status, family income, education, BMI, smoking status, alcohol use, hypertension, diabetes mellitus (DM), hyperlipidemia, and CVD.

Insufficient PA: <600 MET-min/week; sufficient PA: ≥600 MET-min/week.

Low-GNRI: ≤98; High-GNRI: >98.

*P < 0.05; **P < 0.01; ***P < 0.001, indicating statistical significance.

### 3.4 Subgroup analysis and sensitivity analysis

For the subgroup analysis of cancer-related mortality, the high-GNRI group showed significant associations with sex, cancer type, and educational attainment. These associations were more pronounced in the sufficient PA and high-GNRI group, with the following HR and 95% CI: male participants (HR: 0.361; 95% CI: 0.148–0.882), female participants (HR: 0.163; 95% CI: 0.044–0.602), participants with non-obesity-related cancers (HR: 0.236; 95% CI: 0.092–0.604), participants with obesity-related cancers (HR: 0.263; 95% CI: 0.074–0.938), participants with a high school education or equivalent (HR: 0.043; 95% CI: 0.012–0.157), and participants with education above high school (HR: 0.227; 95% CI: 0.097–0.534). The subgroup analysis results for non-cancer-related mortality are provided in Supplementary Table S2. Sensitivity analyses, which excluded participants who died within the first 12 months of follow-up, demonstrated consistent associations between PA, GNRI, and mortality outcomes, further confirming the robustness of the observed effects (Supplementary Table S3).

## 4 Discussion

This study investigates the independent and combined associations of PA and the GNRI with survival outcomes among older cancer survivors in the United States. The findings demonstrate that sufficient PA (≥600 MET-min/week) and High-GNRI (>98) are independently associated with improved survival outcomes. Furthermore, their combination shows a synergistic effect in reducing all-cause and cancer-related mortality. For instance, a 44% reduction in all-cause mortality risk (HR: 0.56, 95% CI: 0.35–0.90) and a 76% reduction in cancer-related mortality risk (HR: 0.24, 95% CI: 0.12–0.50) highlight the potential of targeted interventions to significantly improve survivorship outcomes, providing new evidence for these modifiable factors in cancer survivorship care.

Kaplan–Meier survival analyses confirmed significant differences in survival probabilities across PA-GNRI subgroups, while RCS analyses revealed non-linear associations between GNRI and mortality. Specifically, lower GNRI values were strongly associated with increased mortality risks, but diminishing returns were observed at higher GNRI levels. These trends suggest that excessively High-GNRI values may reflect overnutrition or metabolic imbalances, such as obesity-related inflammation, which could counteract survival benefits. Identifying an optimal GNRI range is therefore critical for maximizing clinical benefits. Future research should explore these non-linear trends and incorporate additional metrics, such as inflammatory markers and body composition analyses, to better understand these relationships.

Previous studies have demonstrated the benefits of PA in improving outcomes for cancer survivors, including reduced all-cause and cancer-related mortality (42, 43). Trials have shown PA positive impact on survival in cancers such as colon, breast, hematological, and endometrial cancers (44–48). Low serum albumin levels in cancer patients may reflect their nutritional status and serve as an indicator of their response to adjuvant therapy, as well as the severity of the underlying disease (49–51). In many cases, hypoalbuminemia is associated with systemic inflammation and tumor progression, which can influence treatment outcomes. Therefore, monitoring serum albumin levels in cancer patients could provide valuable insights into both their nutritional risk and the effectiveness of ongoing treatments. The GNRI, developed by Bouillanne et al., is a validated tool for predicting morbidity and mortality in elderly patients (19, 52). A meta-analysis highlighted GNRI predictive value in head and neck cancer, with low GNRI scores linked to poorer survival (38). Similarly, a study of esophageal squamous cell carcinoma patients found that reduced GNRI was associated with worse outcomes (53). GNRI has been validated as a prognostic marker in various cancers, where better nutritional status correlates with improved survival (28, 52, 54, 55). This study further explores the joint effects of PA and GNRI in cancer survivors, focusing on obesity-associated tumors. Our results indicate that sufficient PA combined with High-GNRI is associated with a 74% reduction in mortality risk among obese cancer survivors (HR: 0.26; 95% CI: 0.07–0.94). By using a nationally representative sample, we confirm PA and GNRI as critical survival predictors. However, while GNRI is practical in clinical settings, it may not capture complex conditions like sarcopenia, suggesting that complementary assessments could enhance its use in cancer survivorship care.

This study has several limitations. The median follow-up of 7.83 years may be insufficient to capture long-term survival impacts, particularly for cancers with slower progression, highlighting the need for extended follow-up. While GNRI is a valuable tool for assessing nutritional risk, it can be influenced by factors such as cancer stage, tumor grade, and comorbidities. These factors can affect both the nutritional status and overall prognosis of cancer patients, potentially impacting the accuracy of GNRI-based assessments. Therefore, the use of GNRI in cancer survivors should be interpreted with caution, especially when these variables are not accounted for. Furthermore, cancer treatments like chemotherapy and surgery can cause weight fluctuations and changes in serum albumin levels, further complicating GNRI interpretation. GNRI should be used alongside other assessments for a more comprehensive evaluation. The GPAQ, while validated, relies on self-reported physical activity, which may introduce recall bias. Healthier survivors are likely to recall activity more accurately, while weaker ones may underreport it. Future studies should consider objective measures, such as wearable activity trackers. Residual confounding from unmeasured factors, such as genetic predispositions or psychosocial influences, cannot be ruled out. Additionally, this exploratory study did not apply multiple testing adjustments, focusing on identifying associations between PA, the GNRI, and mortality outcomes. The cross-sectional design of NHANES linked to mortality data precludes causal inference, emphasizing the importance of prospective studies. Lastly, complete-case analysis for missing data may introduce bias, warranting the use of advanced imputation methods in future research.

These findings offer practical guidance for cancer survivorship care. Tailored PA interventions, such as low-impact aerobic exercises and resistance training, should be prioritized for older adults to enhance adherence and safety. Meanwhile, GNRI can serve as a simple and reliable tool for identifying nutritional risk and guiding timely interventions. However, further validation is needed, especially in regions with differing health behaviors or limited resources. International studies could provide insights into the universal relevance of these interventions. The observed hazard ratio reductions underscore the clinical significance of PA and GNRI, emphasizing their potential to improve survival outcomes and inform evidence-based survivorship care guidelines.

## 5 Conclusion

This study underscores the importance of PA and GNRI in improving survival outcomes among older cancer survivors. Future research should extend follow-up durations, include detailed cancer staging and treatment data, and further investigate the combined effects of these factors. Personalized and accessible interventions targeting PA and nutrition have the potential to significantly enhance the quality of life and survival rates of cancer survivors. By emphasizing accessible interventions, these findings could inform global cancer survivorship care frameworks, particularly in resource-limited settings.

## Data Availability

The original contributions presented in the study are included in the article/Supplementary material, further inquiries can be directed to the corresponding author.
