# Supplementary material for: Joint association of physical activity and the geriatric nutritional risk index with survival outcomes among cancer survivors in the United States: a population-based cohort study
Source: Front Nutr. 2024 Dec 30;11:1483507. doi: 10.3389/fnut.2024.1483507 (PMC11725468; doi:10.3389/fnut.2024.1483507)
Supplement: Supplementary file 1 [file Data_Sheet_1.PDF]

Supplementary files

Joint Association of Physical Activity and the Geriatric Nutritional Risk Index with Survival Outcomes Among Cancer Survivors in the United States: A Population-Based Cohort Study

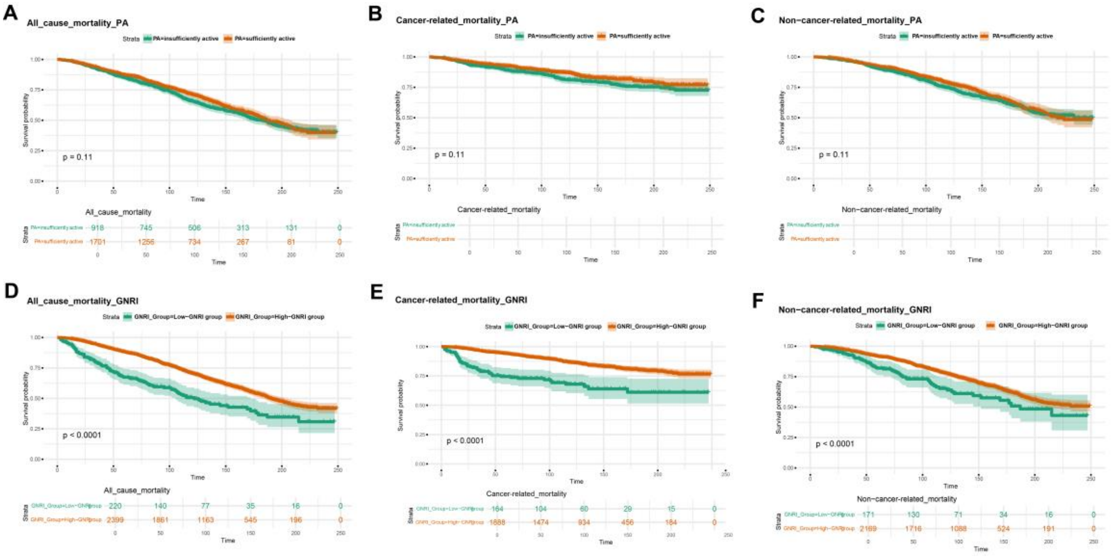

Figure S1. Kaplan-Meier survival curves illustrating the associations of PA and the GNRI with mortality outcomes, including all-cause, cancer-related, and non-cancer-related mortality. A. Survival curves for all-cause mortality by PA levels. B. Survival curves for cancer-related mortality by PA levels. C. Survival curves for non-cancer-related mortality by PA levels. D. Survival curves for all-cause mortality by GNRI levels. E. Survival curves for cancer-related mortality by GNRI levels. F. Survival curves for non-cancer-related mortality by GNRI levels. Insufficiently active PA: <600 MET-min/week. Sufficiently active PA: ≥600 MET-min/week. Low-GNRI: ≤98. High-GNRI: >98. *P < 0.05; **P < 0.01; ***P < 0.001, indicating statistical significance. PA: physical activity, GNRI: Geriatric Nutritional Risk Index.

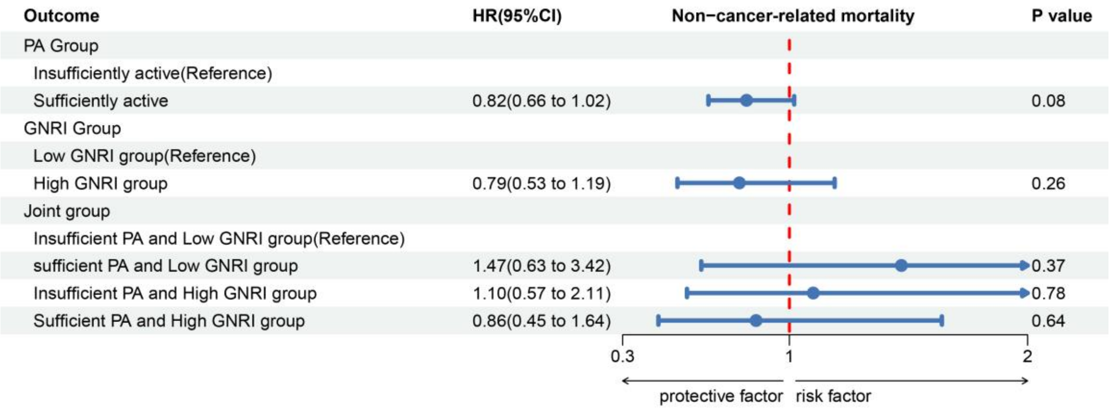

Figure S2. Figure S2. The forest plot illustrates the associations of PA, GNRI, and their combination with non-cancer-related mortality in 2,619 cancer survivors. The results were adjusted for confounding factors, including age, sex, race/ethnicity, marital status, education, household income, body mass index (BMI), smoking status, alcohol consumption, hypertension, diabetes mellitus, hyperlipidemia, and CVD. Insufficiently active PA: <600 MET-min/week. Sufficiently active PA: ≥600 MET-min/week. Low-GNRI: ≤98. High-GNRI: >98. Statistical significance: *P < 0.05; **P < 0.01; ***P < 0.001. PA: physical activity, GNRI: Geriatric Nutritional Risk Index, CVD: cardiovascular disease.

Table S1. Association of PA and GNRI with all-cause mortality, cancer-related mortality, and non-cancer-related mortality among cancer survivors in the United States.

Mortality outcome	crude model		Model 1		Model 2		Model 3	
	HR (95%CI)	P	HR (95%CI)	P	HR (95%CI)	P	HR (95%CI)	P
**All-mortality**								
**PA_Group**								
insufficiently active	ref		ref		ref		ref	
sufficiently active	0.86(0.72,1.03)	0.10	0.77(0.65,0.91)	0.002**	0.80(0.67,0.95)	0.01*	0.82(0.69,0.98)	0.03*
**GNRI_Group**								
Low-GNRI group	ref		ref		ref		ref	
High-GNRI group	0.51(0.37,0.71)	<0.0001***	0.47(0.36,0.62)	<0.0001***	0.53(0.38,0.72)	<0.0001***	0.61(0.45,0.83)	0.002**
**Cancer-mortality**								
**PA_Group**								
insufficiently active	ref		ref		ref		ref	
sufficiently active	0.72(0.54,0.97)	0.03*	0.62(0.46,0.82)	<0.001**	0.66(0.49,0.87)	0.004**	0.67(0.50,0.91)	0.01*
**GNRI_Group**								
Low-GNRI group	ref		ref		ref		ref	
High-GNRI group	0.32(0.20,0.52)	<0.0001***	0.29(0.18,0.45)	<0.0001***	0.34(0.21,0.56)	<0.0001***	0.37(0.22,0.63)	<0.001***
**Non-cancer-mortality**								
**PA_Group**								
insufficiently active	ref		ref		ref		ref	
sufficiently active	0.89(0.72,1.09)	0.26	0.76(0.62,0.93)	0.01*	0.79(0.64,0.97)	0.02*	0.82(0.66,1.02)	0.08
**GNRI_Group**								

Low-GNRI group	ref		ref		ref		ref	
High-GNRI group	0.65(0.45,0.96)	0.03	0.59(0.43,0.81)	0.001	0.66(0.45,0.96)	0.03	0.79(0.53,1.19)	0.26

Abbreviations:

PA: Physical Activity; GNRI: Geriatric Nutritional Risk Index; CVD: cardiovascular disease; HR: Hazard Ratio; CI: Confidence Interval.

Notes:

The crude model represents unadjusted analyses.

Model 1: Adjusted for age and sex.

Model 2: Adjusted for age, sex, ethnicity, family income, education, and marital status.

Model 3: Adjusted for age, sex, ethnicity, marital status, family income, education, BMI, smoking status, alcohol use, hypertension, diabetes mellitus (DM), hyperlipidemia, and CVD.

Insufficiently active PA: <600 MET-min/week; sufficiently active PA: ≥600 MET-min/week.

Low-GNRI: ≤98; High-GNRI: >98.

*P < 0.05; **P < 0.01; ***P < 0.001, indicating statistical significance.

Table S2. The subgroup analysis of PA combined with GNRI with all-cause mortality, cancer-related mortality, and non-cancer-related mortality among US cancer survivors.

**All-mortality_Group**									
**Subgroup**	Insufficiently active PA and Low-GNRI group	sufficiently active PA and Low-GNRI group	p	Insufficiently active PA and High-GNRI group	p	sufficiently active PA and High-GNRI group	p	p for trend (character 2 integer)	p for interaction
sex									0.357

Male	ref	0.887(0.345,2.286)	0.805	0.801(0.410,1.563)	0.515	0.790(0.403,1.546)	0.491	0.461	
Female	ref	0.791(0.324,1.930)	0.606	0.550(0.275,1.100)	0.091	0.390(0.190,0.799)	0.010*	0.002**	
**cancer**_obesity_type									0.673
non-obesity-related cancers	ref	0.873(0.346,2.201)	0.773	0.583(0.311,1.092)	0.092	0.589(0.308,1.123)	0.108	0.105	
obesity-related cancers	ref	0.677(0.240,1.908)	0.461	0.771(0.320,1.855)	0.561	0.565(0.253,1.261)	0.163	0.062	
edu									0.049
Under high school	ref	3.752(0.836,16.841)	0.084	3.229(0.813,12.826)	0.096	1.995(0.529, 7.519)	0.308	0.464	
High school or equivalent	ref	0.886(0.279,2.816)	0.838	0.587(0.263,1.313)	0.194	0.498(0.212,1.168)	0.109	0.046*	
Above high school	ref	0.485(0.208,1.129)	0.093	0.424(0.244,0.736)	0.002**	0.424(0.227,0.793)	0.007**	0.122	

Cancer-mortality_Group

**Subgroup**	Insufficiently active PA and Low-GNRI group	sufficiently active PA and Low-GNRI group	p	Insufficiently active PA and High-GNRI group	p	sufficiently active PA and High-GNRI group	p	p for trend(character 2 integer)	p for interaction
sex									0.579
Male	ref	0.463(0.137,1.563)	0.215	0.387(0.159,0.941)	0.036*	0.361(0.148,0.882)	0.025*	0.058	
Female	ref	0.371(0.091,1.503)	0.165	0.282(0.086,0.919)	0.036*	0.163(0.044,0.602)	0.007**	0.011*	
cancer_obesity_type									0.834
non-obesity-related	ref	0.353(0.102,1.222)	0.100	0.236(0.091,0.613)	0.003**	0.236(0.092,0.604)	0.003**	0.026*	

cancers									
obesity-related cancers	ref	0.440(0.115,1.687)	0.231	0.421(0.117,1.518)	0.186	0.263(0.074,0.938)	0.039	0.036	
edu									0.011
Under high school	ref	5.990(0.532,67.412)	0.147	3.925(0.669,23.021)	0.130	1.868(0.295,11.840)	0.507	0.389	
High school or equivalent	ref	0.167(0.034, 0.827)	0.028*	0.084(0.025, 0.277)	<0.0001***	0.043(0.012, 0.157)	<0.0001***	<0.0001***	
Above high school	ref	0.332(0.124,0.891)	0.029*	0.224(0.096,0.522)	<0.001***	0.227(0.097,0.534)	<0.001**	0.041*	

Non-cancer-mortality_Group

**character**	Insufficiently active PA and Low-GNRI group	sufficiently active PA and Low-GNRI group	p	Insufficiently active PA and High-GNRI group	p	sufficiently active PA and High-GNRI group	p	p for trend(character2 integer)	p for interaction
sex									0.306
Female	ref	0.969(0.349,2.693)	0.952	0.664(0.266,1.656)	0.380	0.477(0.190,1.200)	0.116	0.02**	
Male	ref	1.338(0.446,4.013)	0.604	1.477(0.626,3.486)	0.373	1.436(0.624,3.304)	0.395	0.517	
cancer_obesity_type									0.521
obesity-related cancers	ref	0.648(0.177,2.372)	0.513	1.097(0.350,3.437)	0.874	0.788(0.287,2.165)	0.645	0.335	
non-obesity-related cancers	ref	1.315(0.424,4.081)	0.636	0.886(0.400,1.962)	0.765	0.868(0.388,1.942)	0.730	0.435	
edu									0.124

High school or equivalent	ref	2.332(0.496,10.966)	0.284	1.597(0.583, 4.374)	0.362	1.412(0.487, 4.096)	0.525	0.908
Under high school	ref	4.529(0.769,26.675)	0.095	3.863(0.654,22.839)	0.136	2.252(0.409,12.387)	0.351	0.54
Above high school	ref	0.477(0.143,1.591)	0.228	0.545(0.241,1.235)	0.146	0.525(0.215,1.282)	0.157	0.436

Abbreviations:

PA: Physical Activity; GNRI: Geriatric Nutritional Risk Index; CVD: cardiovascular disease; HR: Hazard Ratio; CI: Confidence Interval.

Notes:

The crude model represents unadjusted analyses.

Model 1: Adjusted for age and sex.

Model 2: Adjusted for age, sex, ethnicity, family income, education, and marital status.

Model 3: Adjusted for age, sex, ethnicity, marital status, family income, education, BMI, smoking status, alcohol use, hypertension, diabetes mellitus (DM), hyperlipidemia, and CVD.

Insufficiently active PA: <600 MET-min/week; sufficiently active PA: ≥600 MET-min/week.

Low-GNRI: ≤98; High-GNRI: >98.

*P < 0.05; **P < 0.01; ***P < 0.001, indicating statistical significance.

Table S3. Sensitivity analysis demonstrating robust associations between PA, the GNRI, and their combination with all-cause mortality, cancer-related mortality, and non-cancer-related mortality among cancer survivors in the United States. (Excludes participants who died within the first 12 months

of follow-up.)

sensitivity analysis	crude model		Model 1		Model 2		Model 3	
	95%CI	P	95%CI	P	95%CI	P	95%CI	P
**All-mortality**								
**PA_Group**								
insufficiently active	ref		ref		ref		ref	
sufficiently active	0.88(0.73,1.05)	0.16	0.78(0.65,0.93)	0.01*	0.81(0.68,0.97)	0.02*	0.83(0.69,1.00)	0.06
**GNRI_Group**								
Low-GNRI group	ref		ref		ref		ref	
High-GNRI group	0.57(0.40,0.79)	<0.001**	0.52(0.38,0.70)	<0.0001***	0.58(0.41,0.81)	0.002**	0.66(0.47,0.92)	0.01*
**GNRI+PA_Group**								
Insufficiently active PA and Low-GNRI group	ref		ref		ref		ref	
sufficiently active PA and Low-GNRI group	0.84(0.46,1.53)	0.56	0.89(0.51,1.57)	0.70	1.02(0.55,1.90)	0.95	0.98(0.50,1.92)	0.94
Insufficiently active PA and High-GNRI group	0.55(0.34,0.90)	0.02*	0.56(0.36,0.90)	0.02*	0.66(0.39,1.11)	0.12	0.73(0.44,1.20)	0.21
sufficiently active PA and High-GNRI group	0.49(0.29,0.83)	0.01*	0.44(0.27,0.72)	<0.001**	0.53(0.31,0.92)	0.02**	0.61(0.36,1.02)	0.06
p for trend(character2integer)		0.002		<0.0001		<0.001		0.01
**Cancer-mortality**								
**PA_Group**								
insufficiently active	ref		ref		ref		ref	
sufficiently active	0.73(0.53,0.99)	0.04*	0.62(0.46,0.83)	0.002**	0.65(0.48,0.88)	0.01*	0.67(0.49,0.91)	0.01*
**GNRI_Group**								
Low-GNRI group	ref		ref		ref		ref	

High-GNRI group	0.40(0.24,0.66)	<0.001**	0.35(0.22,0.57)	<0.0001***	0.42(0.25,0.71)	0.001**	0.46(0.26,0.79)	0.01*
**GNRI+PA_Group**								
Insufficiently active PA and Low-GNRI group	ref		ref		ref		ref	
sufficiently active PA and Low-GNRI group	0.47(0.20,1.10)	0.08	0.54(0.23,1.24)	0.15	0.66(0.28,1.54)	0.33	0.52(0.21,1.30)	0.16
Insufficiently active PA and High-GNRI group	0.32(0.16,0.66)	0.002**	0.34(0.17,0.67)	0.002**	0.44(0.21,0.92)	0.03*	0.41(0.19,0.88)	0.02*
sufficiently active PA and High-GNRI group	0.25(0.12,0.54)	<0.001**	0.23(0.11,0.46)	<0.0001***	0.30(0.14,0.64)	0.002**	0.29(0.13,0.65)	0.003**
p for trend(character2integer)		<0.001		<0.0001		<0.001		0.001
**Non-cancer-mortality**								
**PA_Group**								
insufficiently active	ref		ref		ref		ref	
sufficiently active	0.91(0.73,1.12)	0.37	0.77(0.63,0.95)	0.02*	0.80(0.65,0.99)	0.04*	0.84(0.67,1.05)	0.12
**GNRI_Group**								
Low-GNRI group	ref		ref		ref		ref	
High-GNRI group	0.66(0.45,0.98)	0.04*	0.60(0.43,0.83)	0.002**	0.66(0.44,0.99)	0.05	0.79(0.52,1.20)	0.27
**GNRI+PA_Group**								
Insufficiently active PA and Low-GNRI group	ref		ref		ref		ref	
sufficiently active PA and Low-GNRI group	1.04(0.52,2.07)	0.92	1.14(0.59,2.21)	0.70	1.42(0.66,3.07)	0.37	1.41(0.61,3.29)	0.42
Insufficiently active PA and High-GNRI group	0.72(0.40,1.28)	0.26	0.75(0.44,1.29)	0.30	0.90(0.48,1.72)	0.76	1.05(0.55,2.03)	0.88
sufficiently active PA and High-GNRI group	0.65(0.35,1.18)	0.16	0.57(0.32,0.99)	0.05	0.70(0.37,1.35)	0.29	0.85(0.44,1.62)	0.62
p for trend(character2integer)		0.05		<0.001		0.01		0.08

Abbreviations:

PA: Physical Activity; GNRI: Geriatric Nutritional Risk Index; CVD: cardiovascular disease; HR: Hazard Ratio; CI: Confidence Interval.

Notes:

The crude model represents unadjusted analyses.

Model 1: Adjusted for age and sex.

Model 2: Adjusted for age, sex, ethnicity, family income, education, and marital status.

Model 3: Adjusted for age, sex, ethnicity, marital status, family income, education, BMI, smoking status, alcohol use, hypertension, diabetes mellitus (DM), hyperlipidemia, and CVD.

Insufficiently active PA: <600 MET-min/week; sufficiently active PA: ≥600 MET-min/week.

Low-GNRI: ≤98; High-GNRI: >98.

*P < 0.05; **P < 0.01; ***P < 0.001, indicating statistical significance.
